# Malaria; an Essay on the Production and Propagation of This Poison, and on the Nature and Localities of the Places by Which It Is Produced, at Home and Abroad

**Published:** 1828-01-01

**Authors:** 


### MEDICO-CHIRURGICAL

### REVIEW.

(FIRST NUMBER OF 1828.)

"Nec tibi quid liceat sed quid fecisse decebit
"Occurrat, mentemque domat respectus honesti."

VOL. VIII.] JANUARY 1, 1828. [No. 15.

[NEW SERIES.]

### I.

*Malaria: an Essay on the Production and Propagation of this Poison, and on the Nature and Localities of the Places by which it is produced: with an Enumeration of the Diseases caused by it, and of the Means of preventing or diminishing them, both at Home and in the Naval and Military Service.* By JOHN MACCULLOCH, M.D. F.R.S. &c. Physician in Ordinary to His Royal Highness Prince Leopold. Octavo, pp. 480. Longman & Co. 1827.

THE subject of this Essay has undergone investigation, under various names, ever since the days of Hippocrates—perhaps from a much earlier period. Marsh miasmata—paludal effluvia—morbific emanations—epidemic constitutions—vegeto-animal exhalations—malaria, and other appellations, have been conferred on an invisible, intangible, undefinable—and hitherto unascertainable *something*, which has been only appreciated by its *effects*, but which is totally unknown in its *essence*. By a few visionaries, its existence has been denied, because its substance could not be demonstrated; but all men of sober sense and unbiassed observation have acknowledged, not only the existence, but the terrific power, of this invisible agent. The medical practitioners of our fleets and armies, from the melancholy and extensive experience which they have had of its influence in the production of diseases, where its operations have been on a large scale, bear ample testimony, in their writings, to the tremendous effects of this scourge of the human race; but, we believe that no man has ventured to extend its influence to so wide a circle, or multiply its sources to such an indefinite extent, as Dr. Macculloch. For this he will be deemed a visionary by many—perhaps by most medical men, who give themselves little trouble in such an investigation. In this Journal, he will find a stanch supporter, though not an idolater. We are in the habit of thinking for ourselves—and we believe we have had a field for observation somewhat wider than Dr. Macculloch has had. We shall freely criticise and freely commend—but always under the guidance of what we conceive to be public justice and public utility.

Dr. Macculloch will secretly acknowledge that he has ardently aimed at being *impressive*, and even *eloquent*. In the former object he has often succeeded—in the latter, seldom. With learning, science, and ample command of words, our author has been singularly unhappy in the construction of his sentences—a majority of which terminate as if something were wrong or defective, obliging the reader to reperuse them. Dr. M. will find that an elegant or eloquent sentence must never cost the reader a thought as to its meaning and import. We make this observation, because we think that the want of brevity and perspicacity will greatly circumscribe the range of utility to which this work might otherwise extend. We shall now proceed to notice the chapters in the order of their occurrence in the book before us.

### CHAP. I.—*Introductory.*

Dr. M. observes, that the existence of *malaria* has long been familiar to physicians, and even the vulgar, as the cause of intermittent fevers; but this is of little use, if the one class and the other are ignorant that, to this same malaria are owing the common fevers of summer and autumn, as well as a host of other and unsuspected diseases, as dysentery, cholera, neuralgia in various forms, nervous and dyspeptic affections—and, finally, what may be termed bad health. Dr. M. concludes, that one half, at least, of human mortality is owing to this cause. It does not, however, bear equally hard on all countries—nor at all times in the same country. In England, for instance, the death of a king by marsh fever would now excite some sensation—"yet thus died Cromwell, one among hundreds; the death, indeed, not without note, but its cause not esteemed out of the ordinary course of mortality." In England, observes our author, "that which has been diminished, has not been extirpated." The fens of Lincoln are not the rivals of Walcheren—nor is Romney equally pestiferous as the Pontine Marshes. Still, as travellers, as merchants, as voyagers, and as soldiers, we are interested in the salubrity or insalubrity of all parts of the world, as well as of our own Islands. Dr. M. thinks we may take the average of life among ourselves at 50—in Holland, at 25—in some districts of France, at 22, 20, 18—so little is the chance of life.

"Let us turn to Italy: the fairest portions of this fair land are a prey to this invisible enemy, its fragrant breezes are poison, the dews of its summer evenings are death. The banks of its refreshing streams, its rich and flowery meadows, the borders of its glassy lakes, the luxuriant plains of its overflowing agriculture, the valley where its aromatic shrubs regale the eye and perfume the air, these are the chosen seats of this plague, the throne of Malaria. Death here walks hand in hand with the sources of life, sparing none: the labourer reaps his harvest but to die, or he wanders amid the luxuriance of vegetation and wealth, the ghost of man, a sufferer from his cradle to his impending grave; aged even in childhood, and laying down in misery that life which was but one disease. He is even driven from some of the richest portions of this fertile yet unhappy country; and the traveller contemplates at a distance deserts, but deserts of vegetable wealth, which man dares not approach,—or he dies." 7.

The above is a fair specimen of the better kinds of Dr. Macculloch's style and manner—we shall have occasion to point out some less favourable specimens as we proceed.

The walls of Imperial Rome cannot keep out this enemy of human life. It enters with the Roman into his chambers, and stalks through his streets—nay, "the hour is impending when the Eternal City will cease to be—when it shall submit to that fate, which has been the fate of proud Nineveh, and Babylon, the queen of nations."

Sicily, Sardinia, Greece, are grand seats of this destructive production, "while, in tropical regions, it is to fall by thousands and tens of thousands, the summer harvest of Death walking hand in hand with that of the vegetable world." England herself is far less exempt from malaria than she is supposed to be. Speaking of our participation, as soldiers, in the malaria of other countries, Dr. Macculloch rises into the sublime, or even the terrific.

"It is disease, not the field of action, which digs the grave of armies; it is Malaria by which the burning spirit, fitted for better things, is quenched, and in the coward's bed of death. This is the Destroying Angel, the real pestilence which walks at noon-day; and to which all the other causes of mortality are but as feeble auxiliaries in the work of destruction." 9.

Most of his readers will agree with our author, that "it is *not* the *field* of action which digs the grave of armies." The idea of a *field* digging a *grave* is more incongruous than that of a grave digging a field. If Dr. M. had substituted the *sword* for the *field* of action, the metaphor would have been highly poetical, and without any thing to shock the ear. We think these little incidental criticisms are fairly called forth, when we see a constant attempt at eloquence, and sublimity of ideas.

Dr. M. alludes to a characteristic feature in the moral character of those who inhabit malarious countries—namely, their firm belief in the healthiness of their native air and soil. Walcheren is instanced as an example. We can positively state that the inhabitants of Walcheren did *not* "repel with indignation the charge of unhealthiness which was brought against their beloved birth-place," by our troops on the late fatal expedition. We were there from the beginning till the end of the expedition, and can vouch for the fact, that the inhabitants considered the Autumn of 1809 as a very sickly season among themselves, and remarked that this was the case at intervals of various duration.

Dr. M. offers nothing new, when he undertakes to prove that the existence of a marsh is by no means essentially necessary for the production of febrific miasmata. Every naval and military surgeon knows, and many of them have demonstrated in their writings, that malaria will often arise from ground parched with heat—from the summits of mountains—the rocky bottoms of ravines—and the densest forests. We need not refer to the writings of Lind, Jackson, Blane, Burnett, Johnson, Dickson, Fergusson, Musgrave, and hundreds of others, for proofs of this assertion.

CHAP. II.—*Nature of the Evidences respecting the Production of Malaria.*

In this chapter, our author is conscious that he is "compelled to resort to proofs of some delicacy, and to appeals to an experience, for which, be it received as it may, he must be very often himself responsible." We are disposed to grant him every degree of liberal indulgence on these delicate points. We agree with him that it is a fundamental fallacy in this case, to limit the power of producing malaria to those soils or situations *only*, where intermittent or remittent fevers are found to prevail. Such mistake, we grant, is very common in the minds of "imperfectly educated practitioners," of which, however, the number is rapidly diminishing.

This fallacy set aside, the real conclusion (observes our author) to be drawn is—"that, wherever *remitting* fevers, or fevers of *whatever nature*, that are *not* contagious, as well as dysenteries, are produced, the proof of Summer malaria is as complete as if the same soils had, in Spring, produced ague." With some modifications, we can have little difficulty in subscribing to this doctrine; for we have invariably maintained that all endemic, as well as epidemic fevers, were owing to something emitted from the earth—rather than to things generated in the air. This last is merely the vehicle by which the miasma is suspended or wafted about, like the mineral impregnation which mingles with the water of spring or stream.

The careful observer will often perceive that there are certain determinate places, without any marshes, where fevers are almost annually prevalent; while other places in the vicinity are almost wholly or nearly exempt. In the *former* localities, (Dr. M. avers) some one of the various circumstances of soil, hereafter to be pointed out as productive of malaria, will, on careful inquiry, be found.

A more delicate proof may be drawn, he thinks, from the fact, that some localities are known to be *unhealthy*, as compared with other neighbouring places.

"Thus it is a vulgar remark, that in certain houses or places, a family is rarely without some sickness; or, to use the strong but coarse language in which it is generally stated, 'that the apothecary is never out of the house.' It is almost equally familiar, that families, which had before been healthy, have become the reverse on changing houses or situations; as, in the opposite cases, that they have recovered health by change of residence. Of such facts as these, there is no observer who must not be able to recollect numerous examples." 19.

The laxity of reasoning among medical men, as to the causes of these local peculiarities, is strongly censured by Dr. Macculloch. But he should be merciful, and recollect that all men are not such acute philosophers as himself.

"To anticipate, but no more than is here necessary, what must shortly be said on the subject, if a gravelly soil is healthy, it is because its easy drainage prevents the growth of that particular vegetation which is the cause of malaria; and if a clayey soil is the reverse, it is because, by lodging superficial water, it generates, however partially, those marshy or undrained spots, or wet woods, or moist meadows, which are the sources of this poison, and, consequently, of the various diseases confounded under the vague term unhealthiness." 21.

It is to be remembered, however, that gravelly soils often contain spots generative of malaria; while large tracts of clayey soil are often as dry, and, therefore, as healthy as the most porous ones.

Dr. Macculloch thinks that, if practitioners will attentively observe the phenomena presented in what are called unhealthy situations, they will find either annual fevers, or bowel-complaints, cholera, head-aches, periodical or irregular rheumatism of the face or head, tooth-aches, sciatica, tic douloureux, or other varieties of neuralgia, bilious affections, or some, or many, of what are vaguely denominated nervous complaints, the prevailing disorders of the said localities. And even if these should be absent, or the poverty or other circumstances of the people should prevent their making them known to the physician, still the sallow complexions, the languor, the irritable tempers, or the melancholy character, of individuals thus unfortunately situated, will, Dr. M. thinks, afford the observer sufficient evidence of the operation of malaria on the glandular viscera and general health.

Our author next adverts to a test of a very delicate nature, namely, the liability of persons, who have once suffered from malaria, to become again affected, when exposed to even a very trifling degree of the same. Thus, the sufferers of Walcheren are known to have experienced relapses in various parts of the world, and at various periods, after that fatal expedition. "Hence, therefore, when we find that such a person has experienced a renewal of his disorder, from communication with a place otherwise suspicious from its nature, it offers as convincing a proof as can be desired, that there, malaria is produced or producible." In such situations, the source of the poison may be so circumscribed, as not to affect people in common health; and this negative argument is often offered in favour of the local salubrity. Speaking of the unpleasant task of pointing out sources of malaria, Dr. M. observes.—

"My duty, as it is my design, is to make them known; let *he* (him) who has the power of convincing mankind that they have been in error, and that they are ignorant, undertake the other task. But time effects what man cannot; and hereafter, perhaps, an English gentleman will be as much surprised that his neighbour should dig a sleeping canal before his door, as that his feudal ancestor should have built his castle in a marsh, and inclosed it within a putrid moat.

"To suggest that he who does this is sowing the seeds of disease, that he may reap the fruit of fevers and apothecaries' bills, is to excite the smile of superciliousness or contempt; as he must long yet submit to be the object of both, who would try to convince mankind, that the pond which has been constructed for a few gold fishes, or the river which meanders through the woody valley, is a death-spring of diseases, or that the fevers and the tooth-aches which are the torments of his family, the ailing wife, who is his own torment, and the sciatica which is the torment of his poorer neighbours, are the produce of a few bunches of rushes, or of a splendid display of waterlillies." 31.

We fear that there will be many who will look upon this doctrine as inclining a little to the visionary. Something must be allowed, however, for the style and ardour of language employed by the author. The matter should not suffer on account of the manner. Dr. M. considers, and not without probability, that the obstinacy of chronic diseases is often owing to the repeated application of their cause (malaria) rather than to any change of structure in the organs affected.

Chap. III.—*Soils and Situations most productive of Malaria.*

This chapter is comparatively short, because it treats of localities, which are generally allowed, or, unfortunately, proved to be prolific of malaria. Marshes claim the first place, and salt-water marshes, especially in the warmer countries, are very properly considered by our author—and, indeed, by most other good observers, as equally pernicious with the fresh-water pools. The salt marshes of Dol, in Normandy, are notoriously productive of intermittents, so that scarcely an inhabitant escapes. The same is seen on the French shores of the Mediterranean—in the Adriatic—in Greece—Italy—Sicily—Sardinia—Spain, and a hundred other parts of the world, including both hemispheres of the tropics. Ever since the days of Sir John Pringle, indeed, it has been admitted, that an admixture of salt water with fresh augments the disposition to the putrefactive process, both in animal and vegetable matters. There is little doubt entertained by medical voyagers and observers, that the shores of coasts, and more especially of rivers, washed even by the sea-tide, are amply productive of the poison in question. The banks of rivers where palms and mangroves abound, have been always observed to give origin to bad fevers, fluxes, &c.

"There are few tracts in England more productive of a Malaria, which is even of a virulent nature, than Heron bay and the river banks in general about Reculver, where the water is salt, and the whole is covered twice in the day. The same, indeed, is true of so many parts of England, that the enumeration would be equally tedious and superfluous. Be the truth, however, what it may, in this case, it will be always the most safe belief to adopt the opinion, and to act on it; as the philosophical evil of the error, if it be one, bears no comparison to its value as a practical security." 39.

That woods and jungles, in hot climates, give origin to miasmata of the worst kind, is well known to all medical men; but some doubt may be entertained as to their insalubrity in Europe. Dr. Macculloch, while he suspects the said localities, admits that he has no positive evidence which he is able to bring against them. He thinks there is strong reason to believe that close and wet woods generate malaria in this, as well as in the warmer countries of Europe. Certain woody districts in Sussex and Kent produce both intermittent and remittent fevers—at least there is no other assignable cause. The same may be said of some parts of Hampshire and Essex, as about Epping Forest, for example.

On the other hand, we have positive testimony that lands which were healthy when covered with wood, have become extremely unhealthy when cleared and cultivated. This has been often observed in America. It may be accounted for in several ways. The woods keep off the direct rays of the sun from the wet earth, and the trees may prevent the propagation of the miasmata through the air. When the woods are cleared away, and the earth turned up, the sun powerfully exhales the miasmata, and the winds waft them about in all directions. Reversely, it follows that the planting of trees will sometimes check the production of malaria, by protecting wet lands from the action of the sun. But it requires much circumspection in deciding on planting or clearing grounds with the view of rendering them more salubrious.

"To say that rice grounds are productive of Malaria, is equally to state a fact notorious to the whole world; while the causes, consisting in a succession of inundation and drainage, approximate them in character to swamps and marshes, however obscure the immediate operation of either in producing this poison may be. How extensively Italy suffers from this cause, it is quite superfluous to say; since the mortality in Lombardy, and elsewhere, arising from it, is matter of daily observation, even to the most incurious travellers. And the same is true of Greece and Sicily, as it is generally of Europe, wherever this grain is cultivated." 46.

Such then are the principal and undisputed sources of malaria, in the description of which it was unnecessary to be at all minute.

CHAP. IV.—*Of Soils and Situations less conspicuously productive of Malaria, or as yet unsuspected of it.*

This is the chapter which contains many views of the author which will, in all probability, be disputed by the critical tribe. We shall endeavour, therefore, to make our author as clearly understood as the limits of a review will permit.

To begin with a marsh or swamp. It is supposed that a certain *extent* of such locality is necessary to the production of disease. "This is an error; and it must be classed among the dangerous ones, as being productive of a false security." This is merely an argument as to the plus or minus of a poison. If a large tract of marsh produce a given quantity of malaria, this sum total must be an union of all the portions generated by its parts. And if, as is generally supposed, this poison be the chemical produce of vegetables acting on water, or water acting on vegetables, "then must every plant and fragment of a plant contribute its share to the deleterious substance." Dr. M. also observes that, as there is a certain analogy between malaria and the matter of contagion, and as we know that a quantity of the *latter* which is quite incognizable by the senses, and insensible to every chemical test, will produce its peculiar disease, so we have no reason to doubt the same effects from malaria.

"It would bear that analogy in this point, which it does to contagion in so many others, if a small quantity were as efficacious a poison as a large one; and there are reasons for supposing, practically, that this is the fact, since it is matter of observation, that a minute's exposure to Malaria, a single inspiration probably, and of a poison which must be far more diluted than contagions can ever be in the same circumstances, is sufficient to excite its fever, and, very notoriously, to re-excite it in those who are subject to that morbid sensibility derived from former or habitual fevers." 54.

From this and other matters of fact or observation, our author thinks he is justified in concluding that—"the quantity of malaria necessary to produce its peculiar disease or diseases, is undefinably small, and probably extremely minute."—Although we are inclined to give our author every indulgence in the investigation of such difficult matters, yet we are here forced to dissent from him on a most important and fundamental point. It is universally acknowledged that we are totally ignorant of the nature of marsh or human effluvium, and only know them by their effects. The observation of these effects would induce us to believe that the *quantity* of human contagion—for instance, of syphilis, small-pox, &c. is of little consequence, whereas the *dose* of marsh miasma is every thing. A man may stay twelve hours on the source of a most deadly miasma with impunity; but let him stay four or six hours more, and death will be his lot. This has been proved at Batavia, the Pontine Fens, &c. During the day there is little danger; but in the night, the poison is almost certain of its victim. To the following passage we must also object.

"Could this admit of doubt, or should those who have made no observations, or who are incapable of observing, choose to deny the well-known facts now alluded to as evidence, it would be proved by the great distance to which Malaria travels through the air without losing its poisonous quality. Not to dwell here on examples which must be adduced hereafter, it is quite familiar that from any known and often very limited spot, this poison will proceed through the air, or on the winds, to distances of three or four miles, exciting as much virulence as in its native marsh. This, to quote a familiar domestic example out of hundreds that might be adduced, occurs on the hills of Kent, far from the marshes of Erith, Northfleet, or Gravesend; and it is easy to see that whatever was the body or quantity of Malaria in the original place of its production, or whatever portion of atmosphere it occupied over the few acres by which it was produced, it must often, in such a course, have been diluted to a degree so incomprehensible, that while we can only wonder how it should exist at all as a distinct substance, or a chemical compound, even more must we be surprised that it should be capable of producing its peculiar diseases, with an activity as great, and often greater, than it did at the very point of its birthplace." 55.

How does the above comport with the well-known fact that our ships moored close along the banks of the Scheldt entirely escaped the fever, while the soldiers quartered within half a cable's length of them were all affected with the endemic? The same has been observed in hundreds of other places. We do not say that miasmata are not carried along by the winds; but we do maintain that they are *weakened* by the dilution, in as nearly a ratio to the distance carried as possible. How is Dr. M. certain that the hills of Kent do not generate miasmata, seeing that hills in other parts of the world are not exempt from such productions? Nothing is more common than marshy spots and even stagnant water on the tops or declivities of hills—while at their bases, springs of water and plashy grounds are invariably found. The marshes of Erith, Northfleet, and Gravesend, may not then be guilty of producing fevers on the distant hills of Kent. We doubt whether the following conclusions will be admitted by the professional public, although Dr. M. seems convinced that he has reduced them to a kind of arithmetical certainty.

"The conclusion is obvious; and there is nothing in it which seems to admit of dispute, since it is almost a question of arithmetic. If the produce of a hundred square feet, or acres, or of any scale and number of parts, can, under a dilution of one thousand or ten thousand times, excite disease, then must, in the inverse ratio, the produce of the one-thousandth or the ten-thousandth portion of that space be capable, before dilution, of producing the same effects; or a single blade of grass acting on water (if this be the cause) may be as efficacious as an acre; supposing, of course, that it is actually applied to that part of the body which can suffer from its action." 56.

Dr. M. next proceeds to an analysis of what a marsh or swamp is. This varies much in its obvious aspect, according to the nature of the plants which form its vegetation—these last being considered, however diversified in kind, as mere vegetables, living or dead—since we do not know that the different kinds of vegetables make any difference in the kind of miasmata, though this is not improbable, considering the great variety of elements which enter into their composition, and the different actions which they induce in the animal body. This, however, is all conjecture, and we must trust to future investigation of the subject.

"But to pass from this; the essential character of all marshes and swamps, as far as we yet can decide, is, that the land should be partially inundated, that it should be dry in some places and wet in others, or that pools and dry spots should be intermixed, or that it should be boggy and soft from the mixtures of earths and decayed vegetables with water, or that it should be subject to peculiar alternations of moisture and dryness, sometimes amounting to absolute inundation in the first case." 61.

Dr. M. remarks that the miasma in question is not produced "by the mixture of decomposed and subcarbonized vegetable matter and water, since it is notoriously not produced by dead peaty bogs, or by *peat which carries no vegetation.*" If Dr. M. had been locally acquainted with some of the immense bogs in Ireland and Scotland, he would know that, on their surface, in the Summer time, there is the most luxurious vegetation, most part of which decays and dies in the Winter—yet *there,* an ague or intermittent of any kind is rarely seen. In such localities, then, there is abundance of living vegetable matter in contact with water during the Summer—yet no miasmata are generated. In Winter, the vegetation dies—and still no morbific effluvium is generated. The conclusion which we would draw from this circumstance is this, that, during the *Summer,* the surface of peat bogs is all *life,* and consequently the pabulum of miasmata does not exist—while, *in Winter,* when the decay or death of vegetation takes place, the *temperature* is unfavourable to the production of the said malaria, and thus the people residing on or near these bogs are fortunately preserved from any insalutary influence.

Recurring to the case of marshes, our author labours to shew that this peculiar contact between a living vegetable, or a vegetable in an incipient or somewhat advanced state of decomposition, and water, does take place in many situations that are not marshes, in the popular, or in any sense of the word;—and, consequently, (if this be granted) that a thousand places hitherto unsuspected, are capable of exciting the disorders which result from malaria.

"Now, that this peculiar state of vegetation, not only as to the appearance and character of the soil, but as to the mode of growth and death, and the very nature of the plants themselves, does occur in numerous situations that are not marshes, is the point to be proved, and is a point indeed that will require no proof to almost the most superficial observers; no proof assuredly to botanists, whatever it may to medical men; not often even to the observant inhabitant of the country, whatever it may to the limited man of towns and cities. If the botanist will recognize the spots in question by the nature of the plants which attach themselves to such soils, if the growth of an Iris, an Equisetum, a Hydrocotyle, points out to him what the farmer sees, though less acutely, in tufts of rushes, or traces by the coarseness of the pasture or the canker of a tree, it is the latter who will know every spot of land about him which asks for drainage, where he to whom these pursuits are strange, will seek in vain, even should he, as a physician, be engaged in investigating this very question in a medical view." 64.

It is a popular opinion, he observes, that the rushy pools and petty swamps so common in high moorlands, are innocent. The fact, he avers, is not so. He has seen intermittents in Wales, and at considerable elevations, in those very situations. The following is an example.

"A considerable body of labourers were employed in excavating a pond on a moor of this nature, situated about a thousand feet above the level of the sea; and in the course of the work, within a very short time, nearly one half were incapacitated by the ague." 67.

Dr. M. is convinced that the minute marshy or swampy spots which occur in thousands of low situations, whether on commons, near woods by road sides, or in innumerable other places where they scarcely or never attract notice, are similarly productive of malaria, though their limited range of action generally renders their power in this manner insensible, unless when houses happen to be erected in their vicinity. In how far meadows are capable of producing malaria, he is not completely able to say. Being often intersected by drains and ditches, the miasmata may be generated by these last, instead of by the including land itself.

"I cannot hope to clear this question by an exact definition; but, taking the term in its usual lax sense, it appears unquestionable that there are many tracts of meadow, or of alluvial land, not marshy, and often not intersected by ditches, at least in a conspicuous manner, which are the sources of Malaria all over Europe." 69.

Such, he observes, is the case with the alluvial tracts at the entrances and sometimes at the exits of the lakes of Switzerland and elsewhere, and in places innumerable where there is no proper marsh, nor even an approach to such a character, but where the prevalent diseases must be owing to malaria.

Volney, who could have had no theory to support, has averred, while travelling through America, that every valley in the countries which he visited, produced the fevers of malaria, enumerating among the sources of this poison, not only marshes and woods, but rivers, mill-ponds, &c. Reverting again to the subject of meadow land, Dr. M. observes, that a fruitful source of miasmata may probably be found in "that drying, during Spring and Summer, which follows the moist or wet condition of such meadow lands, as they are left by the Winter rains." A striking example is given in the lands about Fontainbleau, at the junction of the Yonne and the Seine, notorious for the "*fiévre du pays*," so injurious that few escape intermittents or remittents over a considerable tract, for which there is no other ostensible cause but the meadow lands, which are inundated or soaked in Winter, and exsiccated in Summer. Wherever, therefore, the temperature be sufficiently high, he thinks this will be found a productive source of malaria in all countries.

If some great tracts of meadow land in England have been recovered by drainage from a state of marsh, and are now as dry as the ordinary low lands of plains and valleys—and if these localities still produce malaria and its consequences, it is another point of evidence against the salubrity of meadows generally.

" This is true of the meadows which border the Thames, not only beneath London and through their whole extent, but above it; which, though often retaining the name of marshes, because once marshy, are now as dry as the common meadow lands of inland vallies and plains. It appears to be the fact also in many parts of Cambridgeshire and Essex, and, among others, in the vicinity of Waltham Abbey; as it also is in Kent, in the Isle of Thanet, in Somersetshire, in Lancashire, in Huntingdonshire, and far more commonly indeed than it is necessary or convenient to enumerate. Thus it also was, even in the Carse of Gowrie in Scotland, until that great tract of alluvial meadow was brought into universal cultivation; and this may perhaps serve to prove that the meadow land itself, and not the ditches, was the cause, because the latter remain, while the grass has been succeeded by almost universal crops of grain. And it will be found, in confirmation of this, in France and in Flanders, and probably far wider than I now know, that where tracts bordering the same river, or in any other respect exactly similar, whether in soil or situation, are, respectively, cultivated with grain or kept in grass, there the production of fever or of Malaria is correspondent; occupying the uncultivated lands so as to produce what is popularly called the *fièvre du pays*, as if it was a necessary part of the order of things, and flying from those that have been ploughed for a grain cultivation." 75.

Dr. M. observes, that it is a rooted opinion in England that there can be no malaria on the banks of a running stream. As far as mountain torrents are concerned, this is probably true; but, where rivers slowly meander through low grounds, we must not trust to the mere motion of the water. We know, indeed, that the banks of rivers, in hot climates, are truly pestiferous. In those portions of rivers where the tide of the Ocean reaches, as, for instance, along the Thames as far as Richmond, Dr. M. thinks that malaria must be generated by the exposed mud, at each recession of the tide.

"Whatever doubts may still exist as to rivers in general in our own country, in this case, there is no reason whatever to doubt that such streams as the Ouse, the Lee, and all others flowing with similar difficulty through fertile meadows and with a flat vegetable margin, are productive of Malaria, because the diseases which attend it are common in all those situations." 79.

Dr. M. considers those small streams which flow through gentlemen's grounds "almost like artificial canals," bordered by thin and grassy margins, as productive of disease, however the popular opinion may be against such insalubrity. The same observation applies, *à fortiori*, to canals, which are intermediate between a sluggish river and a stagnant pool, their margins possessing, or being capable of possessing, all the essential qualities of a marsh, "as a diminution of their waters may expose mud impregnated with vegetable matter." This, the Doctor observes, is the point which we must always have in view—"it is the analysis of the whole question."

"If it is not putrefying mud, it is the marshy spot, the peculiar vegetation, or death of vegetation, carried on at a certain point of vaccillation between earth and water, which is the generative cause; and, while this may exist in a hundred different characters of ground or situation, and while further it is not essential that bulk or space should be present, it is easy to see that the business of investigation is, in reality, reduced to a very simple principle; for those, at least, who are gifted with the powers of observation and generalization. Let this fact be ascertained by a due examination of any spot, and the probability, at least, of Malaria is established: let it further be ascertained that certain diseases do belong to those situations, taking care also to prove that they are endemic or local, and the fact of its production is determined." 82.

Our author again passes in review the insalubrity of canals, ditches, drains, privies, moats, &c. Speaking of the late fatal endemic which scourged the unfortunate inmates of the Millbank Penitentiary, he says:—"not the slightest doubt ought for a moment to have existed, either with respect to the cause or the disease." A remedy, he remarks, was sought "by letting in that malaria which it should have been the object to exclude or else destroy." The ditches surrounding fortifications afford our author, of course, ample field for supporting his favourite doctrine—especially the fortifications of the low countries. The following passage in respect to lakes, is not devoid of interest.

"But it must also be said in explanation, (a view which is important, as it concerns all waters of this nature, even to pools,) that in France, it is supposed that the Malaria is not solely produced by the vegetating marsh, but is disengaged from the mud which the summer leaves dry, (a fact which I must notice again) and that it also escapes from the bottom and through the water, accompanying the air which is so notedly extricated in those cases. And in confirmation of this, it is said, that while such pools retain a considerable depth of water, or whenever their banks are steep, no Malaria is produced, but that it appears in the reverse cases, or, either on the diminution of the water in depth, or on its retiring from the shores. The same facts, I should observe, have often also been noticed in the West Indies; while a very strong case, illustrating this particular cause, is stated by Senac, in France, where, in a town previously unaffected by fevers, a violent epidemic was produced, in consequence of an unusual evaporation which exposed a large portion of the bottom of a lake. From these facts it is an obvious inference, that in warm climates, at least, whatever may be the case in our own, tranquil or stagnant water is unsafe in any form, and that a vegetating margin is not rigidly necessary to its pernicious qualities; though it cannot be doubted that the evil is materially diminished by cutting off this additional source of Malaria." 98.

It appears that the present alteration in the canal of St. James's Park, is the suggestion of Dr. Macculloch—but the Doctor's fears are far from being hushed by the amendment which he proposed—"since it is notorious for the abundant produce of aquatic plants, causing in Autumn an even insufferable stench."

"Whether the pond in St. James's square also, forming so refreshing a receptable for its statue, claims the same English exemption or not, must be decided by Monfalcon; as I am not courageous enough to think that such an Italian substance as malaria can exist in the center of the English capital." 101.

Dr. M. remarks, that "to prove that mill-dams, though transmitting large streams, ought to be injurious, from the frequently marshy nature of their margins, would be to repeat what has been said before, respecting the *priori* proofs on this subject in general." About the iron district of Glamorganshire, he informs us, there are numerous large mill-dams, for the supply of machinery; "and there is not one of these, in the lower grounds, which is not notoriously attended by the endemic ill-health of all the immediate residents and visitants, consisting in the diseases already mentioned." Of these, neuralgia, he states, is a very common form. These local exceptions to the general health of the surrounding hills and dry places, are peculiarly remarkable, and have attracted the attention of the inhabitants themselves.

As it is incumbent on medical men to attend to medical topography, and thus to put the doctrines of our author to the test of experience, we shall dwell more on localities than we otherwise would have done, in order to excite the attention of our readers to this important subject of investigation. A mill-dam, he says, at Southend, near Lewisham, affords a striking example, though on a small scale, while it is also an instance applicable to fish ponds, and other kinds of still water similarly circumstanced.

"Here the poorer inhabitants in particular, are notedly subject to intermittent as well as autumnal fever, while they bear marks of glandular visceral affections, and are reported to die of the consequences of those disorders. To have seen the fit of intermittent invariably produced in a susceptible individual by an approach to this pond, hundreds of times, and always within a stated distance of time from the approximation, completes an evidence which cannot be controverted." 105.

In farther illustration, Dr. M. instances the valley of the Ravensburn, with the communicating low lands, including the villages of Lee and Lewisham. There is a peculiar physiognomy, he remarks, attached to all such places, which renders it easy to distinguish them.

"I may here add another instance, from the mill dam of a paper mill in Hertfordshire; after the formation of which, the workmen became subject, in a most serious degree, to remittent fevers, which were, before that, unknown; and as the ground in this particular instance resembled that of an ornamental park, as did the water itself, it may suffice to prove what I have advanced on that particular subject; although it would be easy to confirm this by analogous instances adduced from many of the dressed pleasure grounds ornamented by water, which skirt the

Thames, near Walton and Chertsey, and which occur also in a hundred other places: the produce of a well known improving gardener, or else of his progeny; to the demerits of whom, as the sources of an endemic disease of English landscape, far, very far yet from being extirpated, an eruptive contagion blotting our fair island, it is no small addition that they have, in founding ponds which their vanity mistook for rivers, and in converting rivers into Dutch canals, brought the intermittent to our doors under cover of the breeze of the violet, and formed pest houses of fever where we study to retire for coolness from the heats of the autumn. This is to manufacture a Batavia, in defiance of nature; to court disease through deformity and expence; the evil less, it is true, but of the same kind, and incurred as certainly." 106.

The above is another fair specimen of the style and manner of our author, where he aims at being peculiarly impressive through the medium of unconscionable sentences—a single one of which just occupies *a page* of the original work in the foregoing quotation.

Dr. M. here instances a spot in a high, and formerly healthy part of Hampshire, where a clear and quick stream was dammed up, not long ago, for ornament and use. The immediate consequence was, the production of evening mists, before unknown —and the result was, autumnal fevers. Dr. M. never lets an opportunity slip of levelling a sarcasm at the *country apothecary*, though a better acquaintance with that class of medical practitioners would have taught him, that they *think* just as much as, and probably more than, their brethren in towns and cities.

"A French or an Italian *physician* would be at no loss here in deciding; but the *English apothecary*, having no term but typhus for a destructive fever, decides accordingly; never questioning himself as to the origin of the contagion of which he dreams, nor ever recollecting to wonder why it should not spread to the attendants, when the patient is covered with petechiæ; and thus the public goes on, creating more mill-dams, more fish-ponds, more fictitious rivers, and, after the models of Brown, more fevers." 107.

We are not among those who flatter, for base purposes, one class of the profession at the expense of another. There is a mixture of ignorance and intelligence in every class—and all sweeping conclusions, that would represent one class as dolts and another as angels, are necessarily unjust, as well as ungenerous.

Here our author quotes an instance, (doubtless in his own person) where the recurrence of an intermittent fever, in a susceptible subject, was caused repeatedly, "*by merely entering a*

*garden, containing a pond of the fashion of King William's day, dedicated to gold fishes and river gods."*

With such a rare degree of delicate susceptibility, Dr. M. would prove a most valuable miasmometer, and as such, might render essential service to the government, as well as to families, in ascertaining the salubrity or insalubrity of various localities, before they are selected for public establishments or private residences. Dr. M. relates another instance, which happened at Woolwich. There was a small pond occupying an old gravel-pit on the common, close to a house belonging to the late Dr. Hutton, and occupied by General Stehelin —its whole extent being but a few square yards. It was remarked for a long course of years, that the inhabitants of this house were perpetually harrassed with agues; and it was not until this pond was destroyed by improvements in the Common, that the disease disappeared—for ever. Dr. M. has no doubt that the occurrence of ill health, in numerous places where the gravel pits of commons are filled with water, is the consequence of this very cause; and that, in reality, those situations about London, &c. so often selected for the supposed salubrity of their gravelly soils, "are very general, and not less unsuspected causes of ill health." What will the rosy-cheeked inhabitants of Putney-heath, Wimbledon, and Hampstead, say to this? There is no doubt, however, that, in all these places, *agues* have actually shewn themselves this very year (1827); but then the said complaints have prevailed in every county of England, and in almost every street of London. There is something more, in our humble opinion, than stagnant water or decaying vegetables to be taken into account in explaining the production of epidemics. We apprehend that Sydenham was not far wrong, when he suspected an invisible morbific agent, springing from "the bowels of the earth." Still there can be no doubt that the causes forming the subjects of investigation in this Essay, do ordinarily work the effects ascribed to them by Dr. Macculloch.

To the inhabitants of London, our author could easily point out numerous places, even in their own vicinity, illustrating these several causes, but the remarks already made will enable every medical man at least, to investigate the topography of his neighbourhood—his autumnal practice being a good index of the quantum of malaria disengaged from the soil at the time.

It has often been noticed, in various parts of the world, that the breaking up, for the first time, of pasture lands, is attended with sickness. The evidence on this head is as abundant as it is unquestionable. Volney, Nash, and fifty other authorities, might be cited in proof.

"Why this should be the fact, if it cannot be very precisely explained, is not at least more difficult than most of what else belongs to this subject; since there is a quantity of vegetable matter killed, and therefore submitted to decomposition; and it would be well worth the trouble of those whose local situations give them the means, to inquire whether this, and many other analogous agricultural processes, now little suspected, are not the causes of the fevers which sometimes appear in rural situations in such an inexplicable manner, when these cannot be better accounted for by stagnant waters of various kinds, or by such neglected spots as I have here been pointing out. The remark is of value, be the solution what it may; because the remedy will be found in breaking up such lands in June, or in May, if the summer be the necessary period, or, what is preferable, in the middle of winter; since the decomposition will then take place at a time in which experience has shown that Malaria is scarcely generated in our own country, nor indeed, generally, in Europe. In the case of lands recently recovered by drainage, this precaution is peculiarly deserving of attention, because in this case the danger is greatest; and the same is equally true of woods, the mere felling of which sometimes disengages or produces Malaria, as is a much more certain consequence where, as in America, and as I have elsewhere noticed, these woods are broken up for cultivation." 113.

The same observation applies to drainage as to tillage. A swamp may be too wet to produce miasmata—and a certain drainage may just bring it into that state which is peculiarly favourable for the extrication of the unknown poison. This appears to have been the case with the Campagna di Roma, as far as the facts can be ascertained by comparing the different accounts of Italian writers. The most pointed instance, however, is that of the marsh of *Chartreuse*, near Bourdeaux. A succession of bad fevers, before unknown, shewed themselves immediately after the drainage of the above marsh, first in that part of Bourdeaux which lay nearest to the land reformed, and afterwards through the whole of the town. These fevers lasted for many years, and proved so severe in 1805, that 12000 people were affected, three thousand of whom died in five months!

"It is not difficult to understand that a swamp in which the water is so deep as to impede the growth of as many plants as a drier surface would carry, will produce proportionally less of the poison in question; and that a similar diminution or under proportion of Malaria will attend such a tract of land if it should contain many pools or spots divested of all vegetation. In such a case, we can conceive a certain state of drainage, such as to increase the vegetating surface, without being at the same time complete enough to check the production of Malaria; or a small quantity of poisonous marsh might thus become a large surface of wet and noxious meadow land." 115.

Dr. M. touches on the obvious difficulty resulting from the well-known fact that malaria, as ascertained by its consequences, does obtain in some places perfectly dry and gravelly. Our author says that, before we admit this to be a fact, we should be very certain that, in these instances, the malaria is not transported from other places—or that, in such dry places, there are not ditches and drains left from the operations necessary in rendering the ground dry. This does not, in our opinion, clear up the difficulty, as there are unhealthy places where none of the latter exist, and where there is no ostensible source of morbific effluvium within any reasonable distance.

In a chapter respecting some anomalies and inexplicable instances of salubrity and insalubrity, we were rather surprised to read the following passage:—

"But there is one mystery for which I can conjecture no solution, while it rests on great authorities, and while every imaginable circumstance is present that ought to render the land in question one of the most pestiferous spots under the sun. It is a collection of jungles and woods and marshes and rivers and sea swamps, and it is a flat land under a tropical sun, and it is the land of monsoons; and yet it is a land where fevers are unknown. And this land is our new settlement of Singapore. I dare not attempt to controvert such testimony, and must try to believe what I cannot understand: but others may, for aught I know, be inclined to suspect that some favouritism, not perhaps inexplicable, has dictated this report." 138.

We can relieve our author from the dilemma in which this new settlement has thrown him:—Sincapore is *not* a collection of jungles, woods, marshes, rivers, and sea-swamps; but a cluster of high rocky islands, in the midst of an azure sea, and under a sky that is seldom darkened by a cloud. There are no marshes or swamps in the neighbourhood, and the change of the monsoons is not marked by those storms and tornados which occur in other parts of India. The straits of Malacca and Sincapore were always looked upon as the Montpellier of India. Dr. M. may therefore consider that one of the horns of his dilemma is broken off.

Our author next proceeds to notice certain other sources of malaria than marshes or marshy grounds; and among these the putrefaction of vegetable matters takes a leading rank. The first instance quoted is the process of soaking hemp and flax, the offensive nature of which is well known.

"Of pointed facts beyond number, related both in France and Italy, we find in Lancisi, that numerous severe epidemics in the latter country have been traced to these operations, and, among the rest, a noted one at Ferentino, and another at Orvieto which lasted many years. In the former country, out of similarly numerous cases, severe intermittents broke out in the plain of Forez in 1823, after October, (a very rare occurrence,) and were traced to this cause; and we have the assurance of M. Bourges, that it is invariably pernicious, while he describes one very marked case where fevers occurred in a dry, sandy, and otherwise healthy and elevated situation, being regularly renewed with the steeping and drying of the hemp, and disappearing when that season was over." 141.

In Germany, where this manufacture is extensively carried on, it has been often proved that fevers, and those of a very bad kind, are the result :—a fact which, Dr. M. thinks, tends to establish an opinion elsewhere broached, that the nature or severity of fever may be considerably dependent on the nature of the particular malaria, or the quantity in which it is applied. A more accurate train of investigation, he thinks, may probably detect many sources of malaria, of this kind, of which we do not now dream—as, for example, in garden dunghills and the like. He attaches some suspicion to the decomposition of the wood of casks in which water is kept on long voyages, or on shore. He sees no reason to doubt that the wood, in such instances, should generate malaria by its decomposition, as well as the woody elements of herbaceous plants in general. The same suspicion attaches more strongly to bilge water—especially where there is a mixture of various matters in the ship's hold, as, for instance, where there is a leakage of sugar. The writings of Bancroft, Dickson, M'Arthur, and others, have completely established the important fact that fevers of the most malignant kind have been generated by the action of water on various materials in ship's holds.

Among the intricacies and difficulties attending the investigation, our author has appropriately placed the comparative healthiness of ancient and modern Rome.

" The ordinary conclusions of natural history will determine, in the first place, that the site of Rome, as well as the surrounding country, must, at its foundation, have been a tract of woods, lakes, and marshes; and, that such a territory must have been productive of fevers, appears an inevitable consequence. In spite of this, the city flourished and increased, while the surrounding country was also filled with a population distributed in hamlets and villages. The plain of Latium for example, which is now a desert, was, at that time and long after, rich and populous: and thus also the lake of Castiglione, now infamous for its pestilential air, was the seat of a powerful city which long resisted the arms of Tarquinius Superbus. The ancient Latium was situated near a marsh which is now one of the most destructive spots in this district; and the Romans erected baths beyond the Anio, in a place which is, at present, too hazardous even to be visited. The Lago di Giuturna was a favourite spot with the ancient Romans; yet in later times it rendered Castel Gandolfo uninhabitable, and was therefore drained in 1611, by Paul V. In the time of the Volsci there were twenty-three towns and villages in the Pontine marshes, of which Ardea and Lavinium were two. But as it is unnecessary to accumulate more of these specific facts, I shall only further remark, that history confirms what might have been inferred from general considerations, namely, that the country round Rome was in ancient times interspersed with what were called lakes, and which were, in fact, chiefly marshy pools; as must necessarily be the character of accumulated water in a country of such a form and distribution. And these tracts, which were then populous and flourishing, are now uninhabited deserts; although the lakes and marshes have comparatively disappeared, under different attempts at drainage, attended by various success." 165.

In respect to the Everlasting City itself, at that early period, the facts are similar, and the conclusions not less puzzling. The whole site of ancient Rome formed, as can scarcely be doubted, "a focus of malaria and fevers." Yet the city, as well as the surrounding country, increased rapidly in population. Thus the first census by Servius Tullus produced 80,000 citizens capable of bearing arms—while we find that Ardea alone, which now reckons 600 inhabitants, was then able to raise an army sufficient to resist Rome, and also to send a colony to Saguntum. Ostia, now inhabited by a single innkeeper, became a flourishing city soon after its foundation by Ancus Martius. These are some of the puzzling facts which history has left us to unravel. Whether the production or the virulence of the malaria has increased in modern times—or whether the ancient inhabitants had means of resisting its influence which the moderns have not—are the questions which remain to be solved. It cannot, our author remarks, be safely asserted that at any period of the history of Rome, the city and neighbourhood were free from malaria and its consequences—indeed there is strong reason, he thinks, to believe that it was as poisonous then as it is now—"though the apparent effects or the political consequences were less severe."

"It may be thought indeed, that as to some parts of this district, if not to all, the evil has really increased in modern times, not solely from the decay of agriculture arising from that injudicious political management as to corn laws, so often blamed, and from other analogous causes as often discussed, but from geological changes as to the form of the land itself: and of such facts and their consequences, to a certain extent, there seems ample proof. The joint action of the sea and the rivers will, in the case of the Pontine marshes, easily explain a change on this important point, fully adequate to an increase of the evil: and reasoning of an analogous nature may be applied, under modifications, to more inland districts."* 167.

Dr. M. thinks it not unimportant to remark that, as stated by Theophrastus, the plain of Latium was covered, especially towards the sea, by forests of laurel (bay) and myrtle of such a size as to be used in ship-building—" constituting, doubtless, screens to protect the country from the pernicious southern winds, and to check the propagation, if not the production of malaria." Dr. M. thinks the ancients well knew the value of this expedient, and that much of the evil was thereby warded off—hence the sacred character of groves, and the heavy penalties denounced against those who destroyed or injured them. This explanation we cannot help looking on as very fanciful. It is highly improbable that the Romans, at that time of day, knew much of the nature of marsh miasmata, or the means by which these mysterious agents could be counteracted or avoided, notwithstanding the observation of Pliny that groves absorb and destroy mephitic vapours. We think there can be little doubt that great changes in the climate (we mean territorial) of Rome and its vicinity have taken place within the last two thousand years. We see places entirely changed in the degree of their salubrity or insalubrity, within the short space of a few years, of which we may instance Prince of Wales' Island (in the Straits of Malacca) and St. Helena, in the middle of the Atlantic. Both of these places became very unhealthy a few years ago, though previously they were the refuges of invalids on account of their peculiar salubrity.

The first great territorial change in the Roman vicinity appears to have occurred after the invasion of Attila, when the Tiber broke loose, and the Campagna became a marsh. The

* When we contemplate the millions of myriads of human and other living beings which have mingled with their mother dust in Rome and its vicinity, since the days of Romulus and Remus, it is not a very extravagant flight of fancy to imagine that almost every particle of earth under the feet of the present inhabitants, has been once endued with animal life, and therefore that the vegetables on which the Romans feed spring up from, and are nourished by the ashes of their forefathers! There is something in the thought which makes one shudder, and suggests a faint idea that Nature herself affixes an ultimate limit to the congregation and residence of huge multitudes of human beings in any one spot of this earth's surface, however favoured may be the locality in all other respects. The fond aspiration of "ESTO PERPETUA," then, is one of the many " vanities of human wishes," at which Nature smiles, and disperses in empty air!

drainage was renewed under Theodoric; but, on the expulsion of the Goths, this tract was again neglected, and fell back into the same state. Various attempts were subsequently made on the Pontine Marshes, yet with little success. But, whatever may be the difference between ancient and modern Rome, in the degree of insalubrity, there can be no question that malaria prevailed at an early period, as well as at present. Solinus and Dyonisius inform us, that the first settlers were obliged to abandon the Palatine Mount, in consequence of the pernicious exhalations of the Velabrum; and Columella states, that the land near Tusculum, cultivated in the first Punic war, was pestilential—"the malaria of that tract being probably produced by the present Lago di Castiglione." Dr. M. thinks it probable, that the larger proportion of the pestilence described by the Roman writers, were unusually severe visitations of the marsh fever, though it is not impossible that some of them were contagious diseases. Livy informs us that, in the short period of 173 years, viz. from 287 to 460, A. C. there occurred nineteen distinct plagues, none of them at longer intervals than seventeen years, and some lasting two or three years together. This fact alone, says Dr. M. renders it impossible not to conclude, "that the *fever of malaria* must have prevailed then in as great severity as it does at present." We do not see this inference so clearly as our author. The nineteen distinct visitations, with intervals of seventeen years or so, look more like contagious diseases than malarious fevers. Why the long intervals? Why the continuance of an epidemic for two or three years? These are not the characteristics of the modern malaria of Rome. Rome is healthy in the Winter and Spring—sickly in the Summer and Autumn. But when a contagious malady enters a city, or is generated therein, we know not what may be its duration. Dr. M. does not seem to contemplate the possibility of a disease becoming contagious which had arisen from malaria; yet there are few facts in medical science better established than this. It is a phenomenon, too, which explains many things that are otherwise inexplicable.

In the time of the Republic, we have the direct testimony of many writers, as to the existence of malaria. Cato mentions places where it was impossible to live, on account of the badness of the air; and Livy speaks of tertians and quartans. Varro, with more of the jew than the philosopher about him, advises the proprietor of an unhealthy farm to sell it at any price. The early and continued attempts at drainage render it highly probable that one object, at least, was to improve the salubrity of the soil.

Our author hazards a conjecture as to the cause of the great difference in the extra-urbal population in ancient and modern times. He remarks that, in the same soil, and under the same degree of drainage, a tract of land under the plough is less injurious than in pasture or meadows, "whence it is possible, that the greater salubrity of ancient times was an effect of a cultivation, forced or demanded to a greater extent by the superior political condition of Rome at that time." This is extremely probable; while we may bear in mind, that the ancient Roman population was in a state of wealth and power, which would always attract multitudes from the four quarters of the globe to fill up the vacancies caused by disease. Thus, although Egypt was never without its plagues and its fevers; yet a vigorous government and an industrious people contrived to maintain, in spite of them, a condition of population and wealth which has failed only under the more exterminating malaria of Turkish ignorance and despotism. The same reasoning will apply to Venice, which is fast hastening to the same fate. It was long noted, even in modern times, for its peculiar salubrity; but "is now rapidly undergoing a depopulation, in which disease, formerly unknown or unnoticed, is taking its share."

The sixth chapter of the very interesting work under review is dedicated to the consideration of those revolutions and changes which take place with regard to the production of malaria. Many of the topics, however, have been more or less anticipated in the preceding pages. The simplest and the best known case of diminution of malaria, is that which arises from the drainage of marshes, swamps, and fens. To this measure, both governments and individuals have always had recourse—and this is the great change to which we must attribute the improvements of our own island, as well as of many other countries on the Continent. In tropical climates, little has been done in this respect.

"It is from casual reading of various kinds indeed, that we must ascertain the prevalence of fevers and intermittents during the ruder periods of our history; but when we can, by receding upwards from our own time, discover a gradually greater prevalence of such diseases, and when we find the melioration, reversely, following very accurately the progress of agricultural improvement, the whole conclusion appears to be amply justified." 181.

The local instances are innumerable. The agricultural improvements in Lincolnshire, for example, have been closely followed by a proportionate diminution of the diseases of malaria. But, in low countries, the drainage is difficult, and although the insalubrity is lessened, it is not annihilated. This is the case in

Lincolnshire, Holland, and many other places. Indeed, the drainage may obviously be sufficient for the purposes of agriculture, but ineffectual for the eradication of malaria.

The overflowing of rivers reverses the scene, and gives an increase to the production of malaria. And here our author offers a very ingenious and satisfactory reason for one mode of the said increase at the embouchures of rivers. It is this. All rivers bring down more or less materials from the mountains and countries through which they flow. These debris or alluvia are deposited at the mouths of the said rivers, and, by raising the bed of the stream at this place, cause occasional inundations of the neighbouring grounds, and thus lead to the formation of embankments. But the bed of the river still continuing to rise, the adjacent grounds at length become considerably below the level of high water, and then drainage becomes more and more difficult, with a proportionate increase of malaria.

"Thus they tend to raise the water in its bed, and, consequently, to cause it, on any increase, to overflow, still more certainly, the lands around. And as this effect is the very consequence of the embankment, so, at any given point, the bank must be made to keep pace with the rise of the channel, that the restraint may be effectual and constant. Hence as the river becomes more elevated, the ultimate result is the same as if the surrounding lands had been depressed to the same amount: and thus, while the stream which drained them once can drain them no longer, they become, first, meadows, and ultimately marshes. And if, in the former condition, they can still be drained by means of canals and floodgates, this process becomes in time inefficient, and recourse must be had, as in Holland, to lifting machinery." 199.

There are many causes of those revolutions which take place in the salubrity or insalubrity of countries or local districts. Changes in the mutual level of the sea and land, ascribed by geology to the subsidence or elevation of the latter, as connected with the cause of earthquakes, are a fruitful source of the said revolutions. But we need not dilate on these topics. The reader anxious to gain farther information will consult the work itself.

This brings us to a natural division of the investigation, and to the middle of the volume under review. Our analysis could not be conveniently contained in one article, and, therefore, we shall defer our conclusion of it till our next number; when the propagation of malaria—the climates and seasons most favourable to its production—the geography—the nature of malaria —and, lastly, the general effects of this poison on the human constitution, will be fully considered.

We cannot close this article, however, without expressing our admiration of the industry and research by which Dr. Macculloch has collected together a most astonishing mass of information on minute points of medical topography. The manner in which the local descriptions and details are given, induced us to think, that a great proportion of them were collected on the spot by our author in person; and we were not a little surprised to learn from a gentleman, while writing these lines, that Dr. M. has travelled very little indeed. We verily believed that he had traversed half the globe in quest of the materials which he has collected, and that, in Italy, at least, (the emporium of malaria) he had not left a foot of the Campagna di Roma unexplored. This does not derogate from his credit; and we will say, that Dr. M. has travelled round his library to some end. He has there seen more than we have seen in 20 years perambulations round this earth. In our next number, we shall present our readers with a summary of the remaining chapters in Dr. Macculloch's work, which we have no hesitation in recommending to the attentive perusal of the Profession, as a volume abounding in matters equally curious and important.

